# Supplementary material for: Tuning Strain Stiffening of Protein Hydrogels by Charge Modification
Source: Int J Mol Sci. 2022 Mar 11;23(6):3032. doi: 10.3390/ijms23063032 (PMC8950043; doi:10.3390/ijms23063032)
Supplement: Supplementary file 1 [file ijms-23-03032-s001.zip › ijms-1635213-SI.pdf]

Supplementary Materials for

Tuning Strain Stiffening of Protein Hydrogels by Charge Modification

Jie Gu [1,†], Yu Guo [2,†], Yiran Li [1], Juan Wang [1], Wei Wang [1], Yi Cao [1] and Bin Xue [1,*]

[1] Collaborative Innovation Center of Advanced Microstructures, National Laboratory of Solid State Microstructure, Key Laboratory of Intelligent Optical Sensing and Manipulation, Ministry of Education, Department of Physics, Nanjing University, Nanjing 210093, China; 131242017@smail.nju.edu.cn (J.G.); liyiran0628@nju.edu.cn (Y.L.); wangjuannm@163.com (J.W.); wangwei@nju.edu.cn (W.W.); caoyi@nju.edu.cn (Y.C.)
[2] College of Life and Health Sciences, Northeastern University, Shenyang 110819, China; 1901405@stu.neu.edu.cn
* Correspondence: xuebinnju@nju.edu.cn
† These authors contributed equally to this work.

Experimental section：

Materials: The cell line and associated culture reagents included the following: the calcein-AM and PI double staining kit (cat: KGAF001, Keygen, China), the cell culture medium AMEM and DMEM (cat: 310-010-CL and cat: 319-051-CL, Wisent, China), the culture supplement fetal bovine serum (FBS) (cat: 10091148, Gibco, USA), HK-2 and MHCC 97H (Stem Cell Bank, Chinese Academy of Sciences, China).

Swelling ratio and porosity measurements: For the measurements of the swelling ratio, the hydrogel was immersed in ddH_2O for 24 h. Then, the volumes of the initial hydrogel (V_1) and swollen hydrogel (V_2) were measured using the solvent displacement method. The swelling ratio was calculated by the following equation: $(\varepsilon) = V_2/V_1$. For the porosity measurements, the weight of the wet hydrogel was set as W_1, and the weight of the dry hydrogel was set as W_2. The porosity was calculated by the following equation: $(\delta) = (1-W_2/W_1) \times 100\%$. All experiments were undertaken at room temperature.

Cell culture and viability: 97H and HK-2 cells were cultivated in RPMI-1640 medium (Gibco, USA) and DMEM (Gibco, USA), respectively. Ten percent fetal bovine serum and 1% penicillin & streptomycin were added to the medium. All of the cells used in cell viability testing were used before 5 passages.

For the cell viability of cells living on gel, 100 μL gel was prepared in each well of opaque-walled multiwall plates, and then cells were digested from culture dishes. After counting the cells, the concentration of the cell suspension was adjusted to 6×10^3 cells mL^{-1}. Then, 100 μL of

cell suspension was added to each well containing hydrogel. Incubation of plates was performed at 37 °C and 5% CO_2. After 24 hours, the plate was equilibrated to room temperature for approximately half an hour. Fifty microliters of CellTiter-Glo Reagent (Promega, USA) was added to each well. Finally, the luminescence intensity was recorded using a SpectraMax M5e Multi-Mode Microplate Reader (Molecular Devices, USA).

Additionally, calcein AM and propidium iodide (PI) staining were used to evaluate the cell viability of cells living on the gel. The sample preparation was the same as before. After incubation for the desired time, each well of the 96-well plate was washed twice using media described previously. Calcein AM and propidium iodide (PI) (Thermo, USA) dye solution were mixed well and added to each well. The plate was incubated at 37 °C for 30 min. Then, whole-cell images were obtained using an OLYMPUS-IX73 fluorescence microscope (OLYMPUS, USA).

Figures

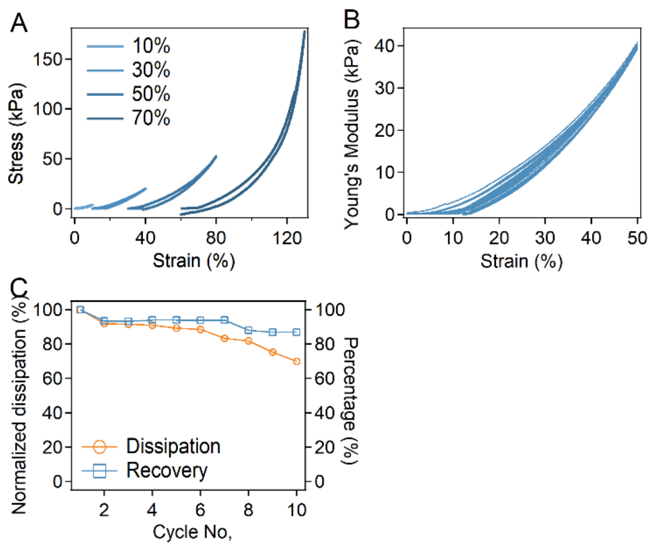

Figure S1 Energy dissipation and recovery of BSA-PEG hydrogels. (A) Stress–strain curves of BSA-PEG hydrogels at different strains. (B) Continuous compression-relaxation cycles at 50% strain to the same BSA-PEG hydrogels for 10 cycles. (C) Normalized maximum stress and energy dissipation corresponding to the same BSA-PEG hydrogels for 10 cycles in (B).

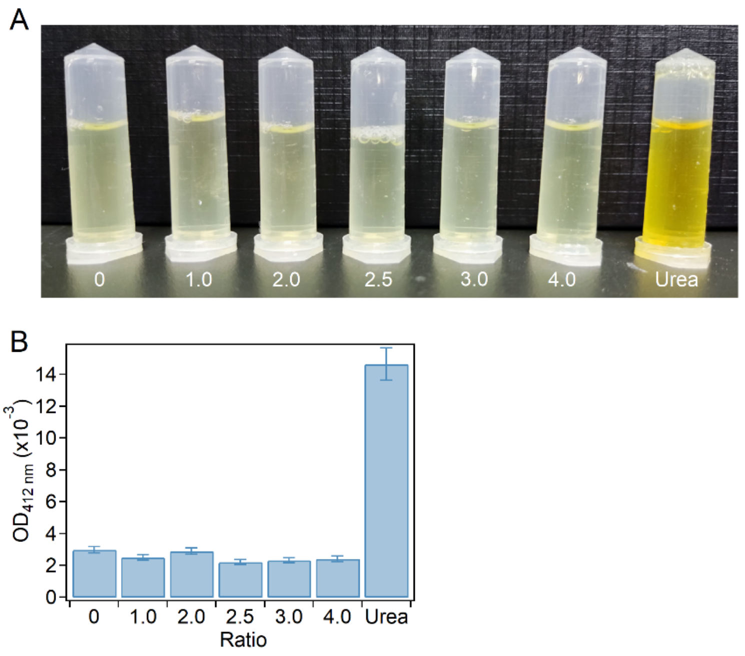

Figure S2 Evaluation of the unfolding of BSA in hydrogels after modification using glyoxylic acid. The unmodified hydrogel treated with 8 M urea was used as the positive control. (A-E) Optical images of thiol detection in BSA-PEG hydrogels modified with glyoxylic acid. Different molar ratios of glyoxylic acid and BSA were used in the modification (0, 1.0, 2.0, 2.5, 3.0 and 4.0). (F) $OD_{412\ nm}$ for DTNB-containing leachates of BSA-PEG hydrogels modified with different equivalents of glyoxylic acid.

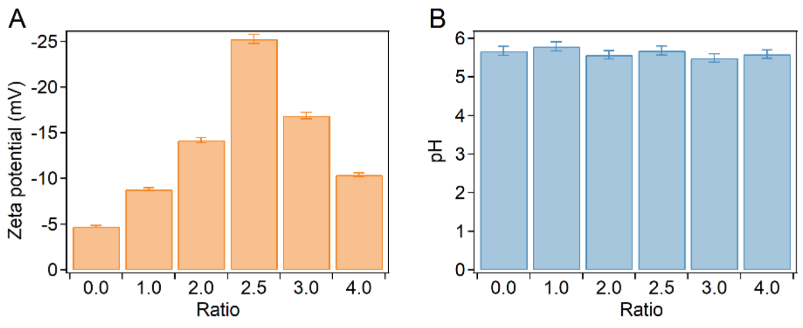

Figure S3 Zeta potentials (A) and pH (B) of the BSA-PEG hydrogels after being split into particles. Different molar ratios of glyoxylic acid and BSA were used in the modification (0, 1.0, 2.0, 2.5, 3.0 and 4.0).

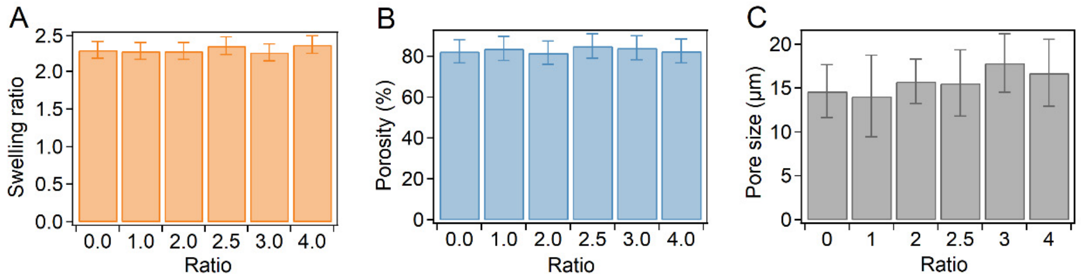

Figure S4 Swelling ratio (A), porosity (B) and pore size (C) of BSA-PEG hydrogels modified with different equivalents of glyoxylic acid (0, 1.0, 2.0, 2.5, 3.0 and 4.0).

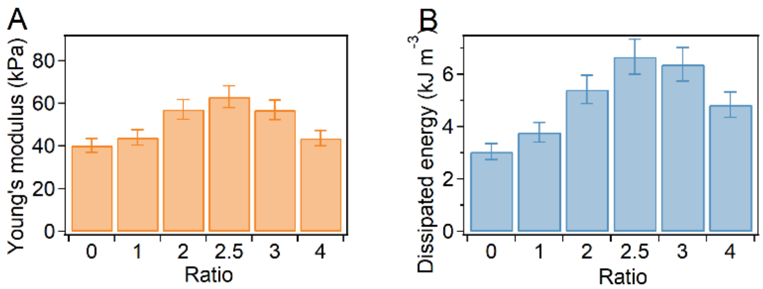

Figure S5 Young's modulus at the strain of 0-10% (A) and dissipated energy at the strain of 0-50% (B) of BSA-PEG hydrogels modified at different ratios of glyoxylic acid and BSA (0, 1.0, 2.0, 2.5, 3.0 and 4.0).

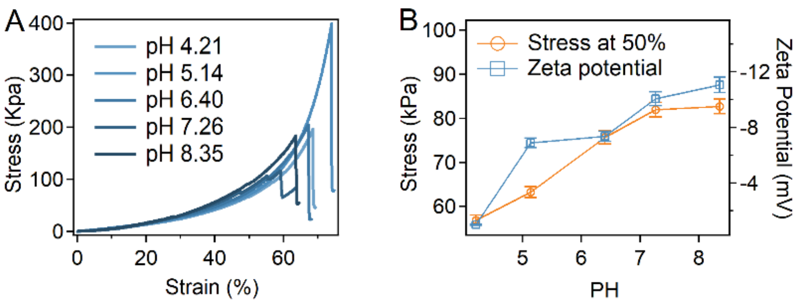

Figure S6 Mechanical properties of unmodified BSA-PEG hydrogels at different pH values. (A) Compressibility of the BSA-PEG hydrogels at different pH values. (B) Stress at 50% and zeta potential of

BSA-PEG hydrogels at different pH values.

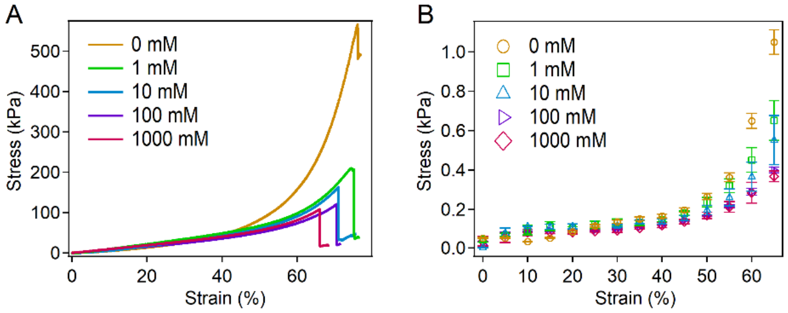

Figure S7 Mechanical properties of unmodified BSA-PEG hydrogels at different salt concentrations (NaCl solutions, 0-1000 mM). (A) Compressibility of the BSA-PEG hydrogels at different salt concentrations (NaCl solutions, 0-1000 mM). (B) Differential modulus corresponding to BSA-PEG hydrogels at different salt concentrations (NaCl solutions, 0-1000 mM).

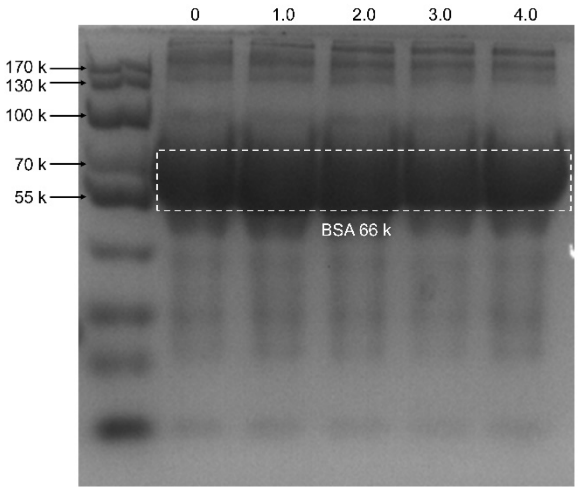

Figure S8 SDS-Page gel of BSA modified at different glyoxylic acid:BSA ratios.

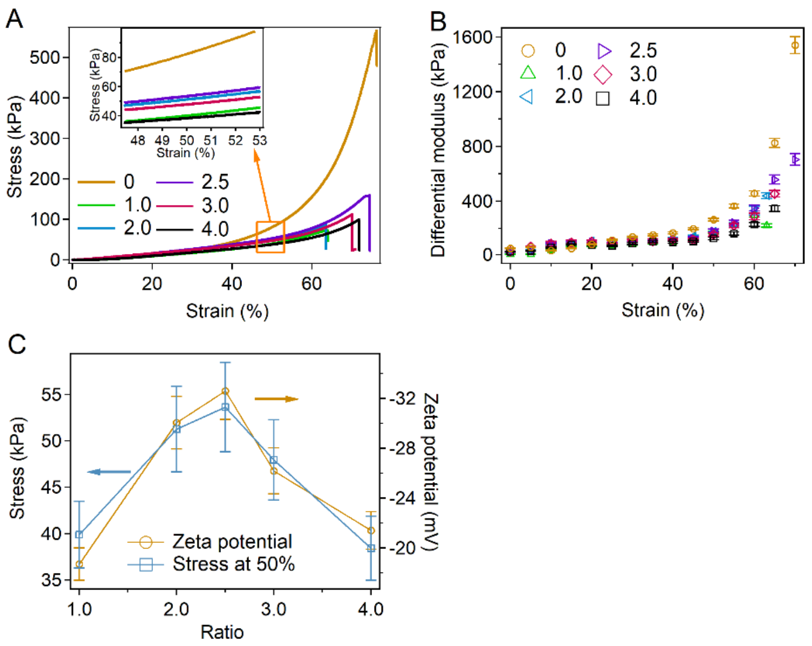

Figure S9 Compressibility of the BSA-PEG hydrogels prepared using BSA modified with glyoxylic acid. (A) Compressibility of BSA-PEG hydrogels prepared using BSA modified with glyoxylic acid. Different molar ratios of glyoxylic acid and BSA were used in the modification (0, 1.0, 2.0, 2.5, 3.0 and 4.0). (B) Differential modulus corresponding to BSA-PEG hydrogels prepared using BSA modified with glyoxylic acid. (C) Summarized stress at the strain of 50% for BSA-PEG hydrogels prepared using modified BSA and zeta potentials of BSA modified at different ratios of glyoxylic acid and BSA.

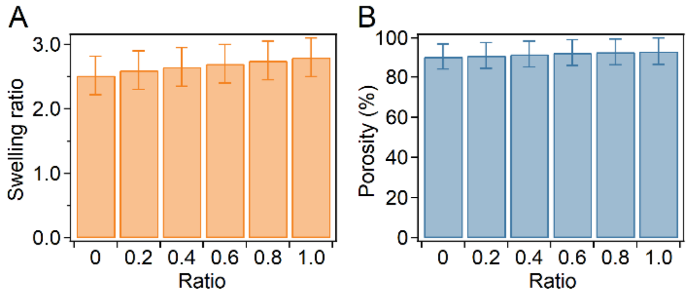

Figure S10 Swelling ratio (A) and porosity (B) of BSA-PEG hydrogels modified at different ratios of bromoacetic acid and BSA (0, 0.2, 0.4, 0.6, 0.8 and 1.0).

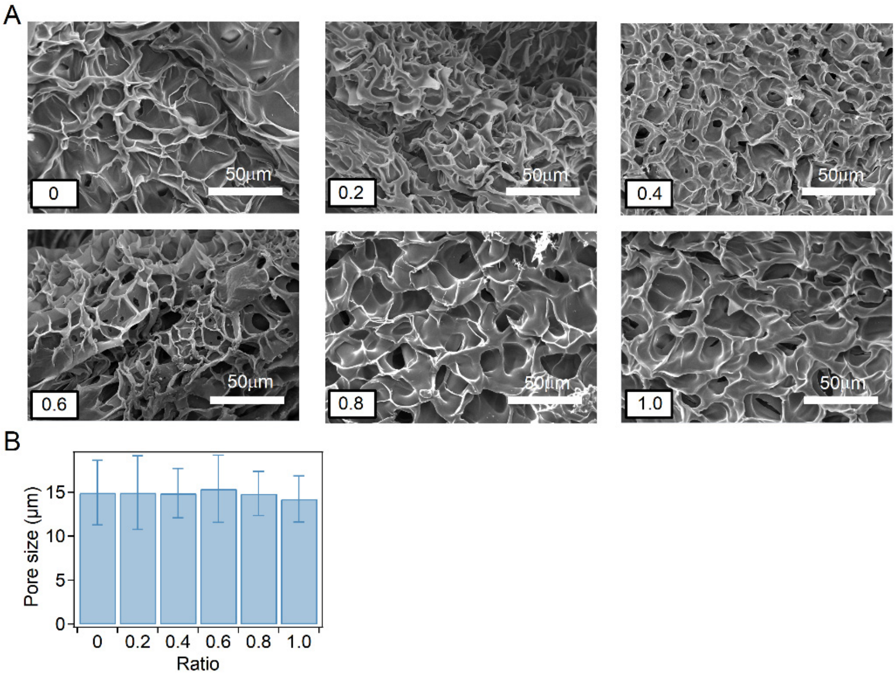

Figure S11 SEM images (A) and pore size (B) of BSA-PEG hydrogels modified with different ratios of bromoacetic acid and BSA (0, 0.2, 0.4, 0.6, 0.8 and 1.0).

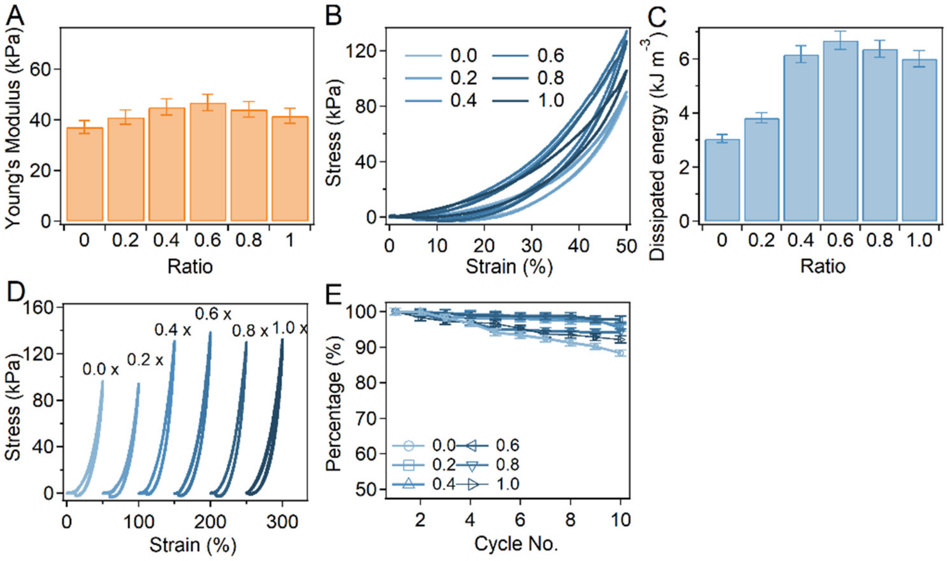

Figure S12 Mechanical properties of BSA-PEG hydrogels modified at different ratios of bromoacetic acid and BSA. (A) Young's modulus of BSA-PEG hydrogels modified at different ratios of bromoacetic acid and BSA. (B) Compression-relaxation cycle of modified BSA-PEG hydrogels at a strain of 50%. (C) Dissipated energy at a strain of 50% for BSA-PEG hydrogels modified with different ratios of bromoacetic acid and BSA. (D-E) Continuous compression-relaxation cycles (D) and normalized maximum stress (E) of BSA-PEG hydrogels modified at different ratios of bromoacetic acid and BSA for 10 cycles.

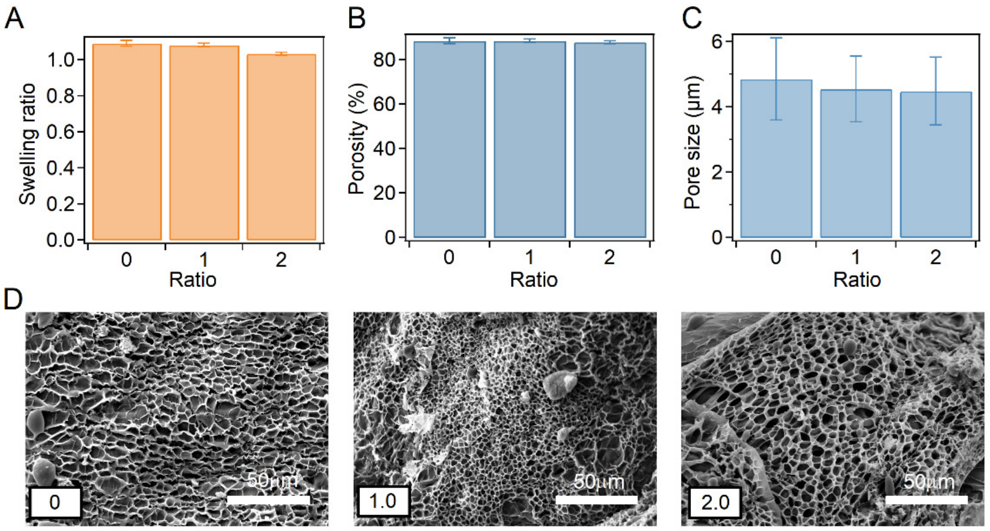

Figure S13 Swelling ratios (A), porosity (B), pore size (C) and SEM images (D) of hemoglobin-PEG hydrogels modified with glyoxylic acid.

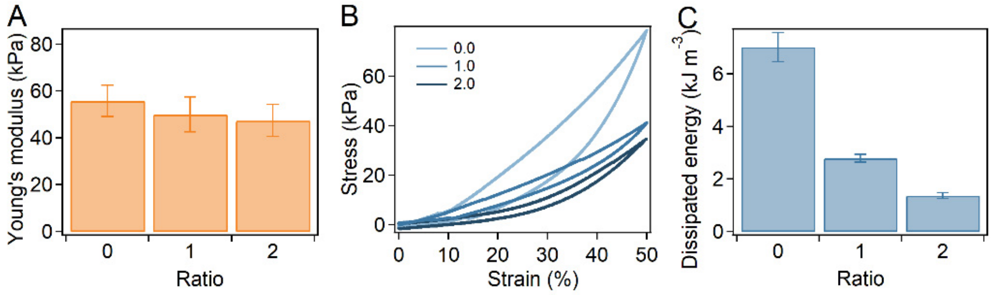

Figure S14 Mechanical properties of hemoglobin-PEG hydrogels modified with glyoxylic acid. Different glyoxylic acid: hemoglobin ratios were used in the modification (0.0:0, 1.0:1 and 2.0:1, simplified as 0, 1 and 2). (A) Young's modulus of hemoglobin-PEG hydrogels modified with glyoxylic acid. (B) Compression-relaxation cycle of modified BSA-PEG hydrogels at the strain of 50%. (C) Dissipated energy at the strain of 50% for hemoglobin-PEG hydrogels modified at different ratios of glyoxylic acid and hemoglobin.

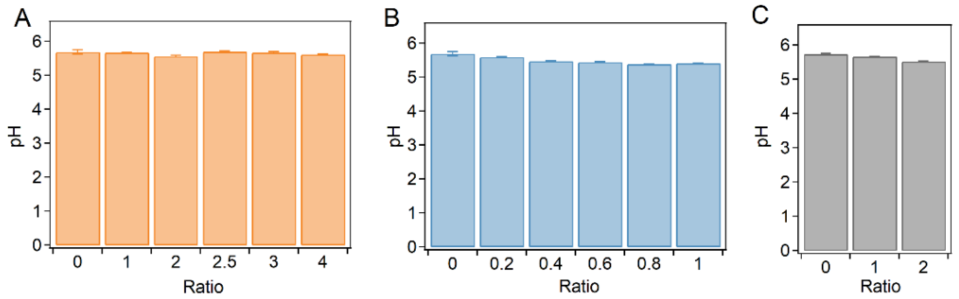

Figure S15 Summary of the pH for the protein solutions after modifications. (A) Summarized pH of the BSA solutions after modifications of BSA using glyoxylic acid. (B) Summarized pH of the BSA solutions after modifications of BSA using bromoacetic acid. (C) Summarized pH of the hemoglobin solutions after modifications of BSA using glyoxylic acid.

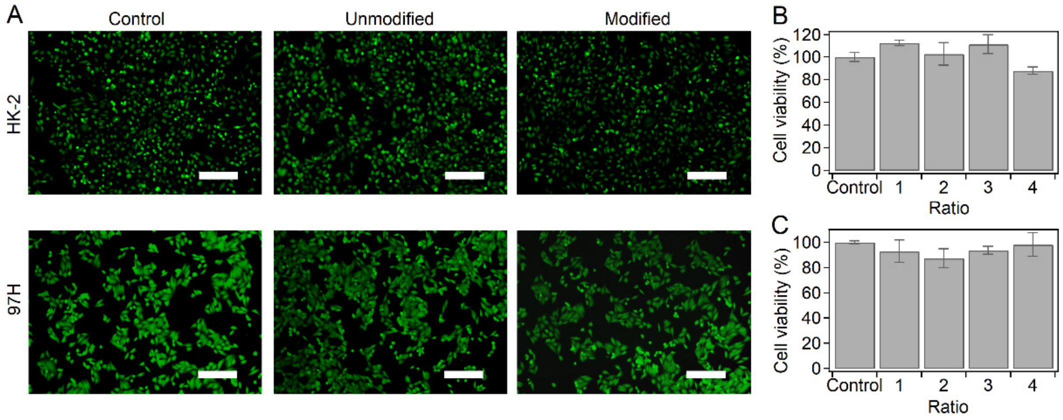

Figure S16 Cell culture and viability on BAS-PEG hydrogels modified using glyoxylic acid. (A) Images of stained cells living on petri dishes (Control), unmodified BSA-PEG hydrogels (Unmodified) and modified BSA-PEG hydrogels (Modified) at the glyoxylic acid:BSA ratio of 2.0. Green represents live cells with high enzymatic activity indicated by calcein AM. The red color of PI represents dead cells with compromised membranes. Scale bar = 100 μm. (B-C) Cell viability determined using Promega CellTiter-Glo for HK-2 cells (B) and 97H cells (C) after being cultured for 24 hours on modified BSA-PEG hydrogels.
